# Perception of Affordances for Dribbling in Soccer: Exploring Children as Architects of Skill Development Opportunity

**DOI:** 10.3390/sports10070099

**Published:** 2022-06-24

**Authors:** Michael J. Duncan, Ricardo Martins, Mark Noon, Emma L. J. Eyre

**Affiliations:** Centre for Sport, Exercise and Life Sciences, Coventry University, Coventry CV1 5FB, UK; marti234@uni.coventry.ac.uk (R.M.); aa5349@coventry.ac.uk (M.N.); ab2223@coventry.ac.uk (E.L.J.E.)

**Keywords:** motor competence, motor skill, youth, grassroots, talent development, mixed methods

## Abstract

This study examined affordance perception for soccer dribbling using a mixed-methods approach in male grassroots soccer players. We examined how children construct and perceive skills practices for dribbling in soccer. Fourteen boys aged 10–11 years (Mean ± SD = 10.8 ± 0.4 years) who were regularly engaged in grassroots soccer participated in the present study. Children were provided with ten soccer cones and asked to create their own soccer dribbling pattern that would enable them to maximise the number of touches with a football and then dribble the ball in the pattern they had created for a 1 min period. Children were interviewed to explore their perception of affordances for soccer dribbling. The test of gross motor development-3 was used to assess fundamental movement skills (FMS), and the UGent soccer dribbling test was used to assess soccer dribbling skills. Children self-rated their own ability for soccer dribbling, as did their coaches. Pearson’s correlations were employed to examine the associations between quantitative variables, and thematic analysis was used to explore qualitative data. Results of the present study suggest that those children who created patterns with less space between cones accrued more touches of the football in their dribbling task (r = −0.671, *p* = 0.03). Children with a higher perception of their own dribbling ability had higher scores for FMS (r = 0.604, *p* = 0.049). Those children who scored better in actual soccer dribbling had higher scores for FMS (r = −0.746, *p* = 0.012) and were rated as better dribblers by their coaches (r = −0.67, *p* = 0.03). Interview data suggest a feedback loop between perception of ability and actual ability, which influenced the dribbling patterns that were created. This suggests that dribbling performance is scaled to the (perceived) action capabilities of the children, and children can act as architects in their own skill development.

## 1. Introduction

The concept of affordances is well established in the sport and exercise science and sports coaching literature [1,2]. Affordances refer to opportunities for action that are available to a given individual in a given environment [2]. There is considerable research, which demonstrates human sensitivity to affordances [1]. Such research is varied, focusing on a range of different movements from sitting on a chair [3], crossing the street [4], using playgrounds [5], scoring in soccer [6], as well as kicking for distance and precision [7]. Through development, a child’s varied movement contexts provide different opportunities or affordances for action that are fundamental to promoting motor competence and sport skills [8]. In the context of sport, perception of affordances and opportunities to develop such perceptions are useful for talent development [9,10]. 

In soccer, there is good evidence that manipulation of constraints can promote perception of affordances, and subsequently, more effective skill development, as a player’s ability to adapt their perception and action to continuous changes in spatial and temporal information in a game underpins successful performance [11,12]. Soccer performance and the execution of motor skills within soccer are complex, comprising technical, tactical, physical and psychological aspects, which collectively contribute to decision making to execute motor skills at an opportune time to result in a successful outcome. Dribbling, passing and shooting are recognised as the most important motor skills in soccer [13] and are consequently a focus for coaches working with children at grassroots levels. The ability to dribble the ball and go past the opposing players is a particularly fundamental aspect to the game of soccer [14] and is a complex skill where players must apply cognitive, perceptual and motor skills to rapidly changing situations when dribbling with the ball to maximise their performance during the game [15]. However, while studies have specifically examined affordances for passing in soccer [16,17], shooting [6] and kicking for distance and/or precision [7], few studies have examined affordances for dribbling in soccer. In the context of assessment, the assessment of dribbling skill has been criticised [14], as the majority of the methods available are based on travelling with a ball around cones placed 2–4 m away from each other in a figure-of-eight movement pattern or similar [18,19,20]. Such a process also translates to how dribbling skill is trained in a blocked and constant manner, where there is repetition of dribbling around cones placed a set distance apart [21,22].

This standardisation of approach does not necessarily translate to an optimal way to coach, assess and improve sport-specific motor skills. Motor skills primarily develop when skills are performed in different ways [23]; perceptual sensitivity to affordances also changes when an individual acquires better motor skills [24]; and standardisation, where distances between cones tend to be equal, does not facilitate variability of practice [5]. Instead, the standardised approach invites the child to continuously replicate a movement pattern. Crucially, affordances exist by virtue of the relationship between the physical properties of the world and the action capabilities of the individual [2]. Whether a space affords the development of soccer dribbling depends on the size of the space, the cones/obstacles/other performers in that space and the rules for use of that space, relative to the skill capabilities of the performer themselves. This means that the same space and constraints can afford different behaviour to children with different action capabilities. Understanding how children may use the same space differently in relation to soccer skill development, and the extent to which other factors, such as perception of their own competence or technical skill, might impact how they use such space, have not been fully examined. Likewise, to date, research has not examined how effective children are as architects of their own soccer skill development. Such information and understanding are useful for coaches in better structuring practices to maximise skill development. The current study sought to explore this issue by examining affordance perception for soccer dribbling using a mixed-methods approach in boys who play grassroots soccer. Using an experimental paradigm, followed by a qualitative contextualisation, we sought to uncover a more in-depth understanding of perception of affordances for dribbling in soccer. We hypothesised that children who perceived themselves as better dribblers would create more challenging dribbling patterns and would also accrue a greater number of touches on the ball in the dribbling pattern they created.

## 2. Materials and Methods

### 2.1. Participants

Fourteen boys aged 10–11 years (Mean ± SD = 10.8 ± 0.4 years, 145.7 ± 3.7 cm, 37.0 ± 4.1 kg) who regularly played organised grassroots soccer for clubs in England participated in the study following institutional ethics approval (protocol code 131207), informed parental consent and child assent. We employed the Fédération Internationale de Football Association (FIFA) [25] definition of grassroots soccer in the present study. To be eligible for participation, children had to be registered/playing with a grassroots soccer club, including participation in training and fixtures against other teams within England’s County FA structure. Participants also had to have at least 1 year of playing experience prior to taking part (Mean ± SD playing experience was 4.2 ± 1.0 years). 

### 2.2. Design and Procedure

An exploratory mixed-methods approach was employed. All assessments took place over two days and were conducted on an artificial football pitch surface. On the first day, anthropometric assessment was conducted, followed by child self-rating of dribbling skill, as was technical skill and general motor competence assessment. This was followed on the second day by the dribbling affordances task and qualitative interview. All assessments were conducted by trained researchers, and the participants’ soccer club coaches were not involved in any way.

### 2.3. Anthropometry

Stature (cm) and body mass (kg) were assessed to the nearest 0.1 cm and 0.1 kg using a SECA anthropometer and weighing scales (SECA Instruments Ltd., Hamburg, Germany), respectively.

### 2.4. Child Perception of Dribbling Ability

Children’s perception of dribbling ability was determined by asking each child individually to rate, on a visual analogue scale, how good they considered their football dribbling ability to be. Each child was asked to score themselves from 0 to 10, with 0 being not able to dribble at all and 10 being able to dribble confidently in a diversity of situations. Each child completed their perception of dribbling ability on an individual basis, prior to any assessment of movement competence or dribbling ability, and followed processes previously employed in the assessment of perceived soccer competence [18] and affordance in gap-crossing in children [5].

### 2.5. Motor Competence Assessment

Motor competence was assessed using selected skills from the test of gross motor development-3 (TGMD-3) [26]. To provide a balance of both locomotor and object control skills, children were assessed on the run, jump, hop, overhand throw, underarm throw and catch skills. The kick skill was excluded to avoid confounding the assessment of motor competence and technical soccer skills. Skill mastery on the TGMD-3 requires each component of each skill to be demonstrated, and each skill comprises 3–5 components. Trials of each skill were video recorded (Sony Handicam CX405b, Sony, Tokyo, Japan). Skills were subsequently edited into individual movie clips using the Quintic Biomechanics software v21 (Quintic Consultancy Ltd., Birmingham, UK). As per TGMD-3 guidelines [26], scores from the two trials were summed (scored 0–50) to create an overall score reflecting total FMS. 

### 2.6. Technical Skills

Soccer dribbling skill was assessed using the Ghent University (UGent) dribbling test as previously described by Vandendriessche et al. [27]. All testing was completed with a size 4 football (the official ball size for age band U10–12) as recommended by the Football Association. Testing was completed individually by the participants to minimise any peer pressure to perform.

Participants completed a set circuit with four left and four right turns at different angles, with a distance between cones ranging between 1 and 2.2 m [27]. Following familiarisation and a practice trial, each participant undertook two attempts at the test. Each test was performed as quickly as possible in two steps per test; the first step was performed without the ball and the second step with the ball. The time of each attempt was measured to the nearest 0.01 s with a handheld stopwatch. The time taken to complete the dribbling course without the ball was deducted from the time with the ball to give a skill differential reflecting the dribbling skill. This test has a good reliability, shown by an intra-class correlation coefficient (ICC) of 0.81 [28] and an ICC of 0.82 in a subsample (*n* = 30) of the current sample [28].

### 2.7. Affordances for Dribbling Task

The affordances for dribbling task was designed based on procedures previously employed in assessing affordances for playground play in children by Jongneel et al. [5] and Sporrell et al. [29]. The affordances for the dribbling task took part in two phases. In the first phase, each child was asked to create a space/formation to maximise their own soccer dribbling. To this end, each child was provided with 10 cones and a space measuring 9 m length by 5 m width. The cones were standard mini soccer cones that were circular, 12 cm in diameter and 4 cm high. Each child was instructed to create their own space that would allow them to have the most touches on the ball within a 1 min period. The child could place the cones wherever they wanted in the space and could use a maximum of 10 cones. To experience whether the constructed space was in line with their desires, the child could move in and around the space as they wished during this phase. Following this, the children were allowed to adapt the course during this phase. Three of the children adapted their course following the trial, making minor changes to placement of a cone. After the child completed the first phase, the formation of cones, trial and adaption of the cones, the distances between cones were measured with a measuring tape. In the second phase of the task, each child was asked to dribble the soccer ball in the space they had created for a period of 1 min. The child’s playing behaviour was video recorded (Sony Handicam CX405b, Sony, UK) and, based upon this, we determined how many touches of the ball were made, with what foot and what patterns of movement occurred in the space.

### 2.8. Qualitative Interviews

Once each child had completed the affordances task one to one, interviews were held with each child in a comfortable room that the child was familiar with. The interview used a structured guide with open questions, images and videos of their and other players’ drills created to use the probe memory. The interview asked 4 main questions and was based on recommendations for conducting qualitative research and validation of interview scheduling [30]: 1. (show their pattern) Why did you create this patten to get the most touches?; 2. How do you feel about the drill you created versus the drill created by your coaches to get the most touches they created versus ones created by their coaches?; 3. (show player’s drills). How do you feel about this drill and the number of touches you would be able to complete doing this drill?; 4. Perceptions of their dribbling ability. Prior to all interviews, the interviewer followed the eight-stage interview preparation stage identified by McNamara [30], which included: 1. Choosing a setting to minimise distraction; 2. Explaining the purpose of the interview; 3. Addressing confidentiality; 4. Explaining interview format; 5. Stating the length of interview duration; 6. Providing researcher contact information; 7. Asking if participants had any questions before starting; and 8. Asking for permission to record their views during the interview. The interview was led by one facilitator. Probing was used throughout the interview to gain further understanding. 

### 2.9. Analysis

Descriptive statistics (mean ± SD) were calculated for touches/min, child perception of dribbling ability, UGent dribbling test score, total FMS score, coach rating of dribbling ability and the average distance between cones in the pattern the child had created. Pearson’s product moment correlations were then used to explore relationships between these aforementioned variables. The Statistical Package for Social Sciences (SPSS V25) was used for all analyses.

### 2.10. Interview Analysis 

Interviews were transcribed verbatim during the interviews. Each participant was anonymised and given a code from 1 to 11. Transcripts were analysed using inductive analysis following the steps proposed by Braun and Clarke [31], such as familiarisation of data, reading and re reading the data, code generation, categorisation, search and reviewing themes and defining and naming themes. This process resulted in themes, sub themes and their associated quotes. This enabled a broad flexible approach for the analysis of the data collected to produce an enriched and detailed account of the findings [31]. Analyst triangulation was conducted to increase the quality and credibility of the findings [32,33] using a second independent analyst who conducted a thematic analysis, which was then compared with the primary researcher, assessing the potential selective perception and blind interpretive bias [32,33]. Frequent de-briefing sessions between authors facilitated the discussion, debate and re-definition of the themes. Following completion of the coding process, pen profiles were created to help present the emergent themes via diagrams with the number of times the themes were mentioned [34]. 

## 3. Results

Mean ± SD of touches/min, child perception of dribbling ability, time taken on the UGent dribbling test and total FMS score are presented in Table 1.

**Figure 1:**
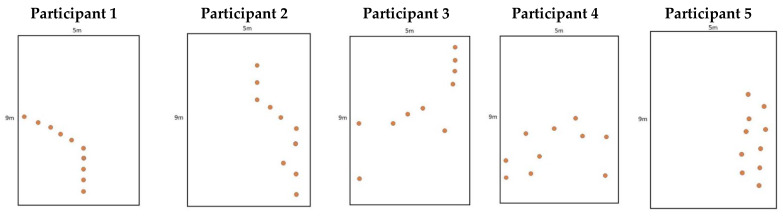

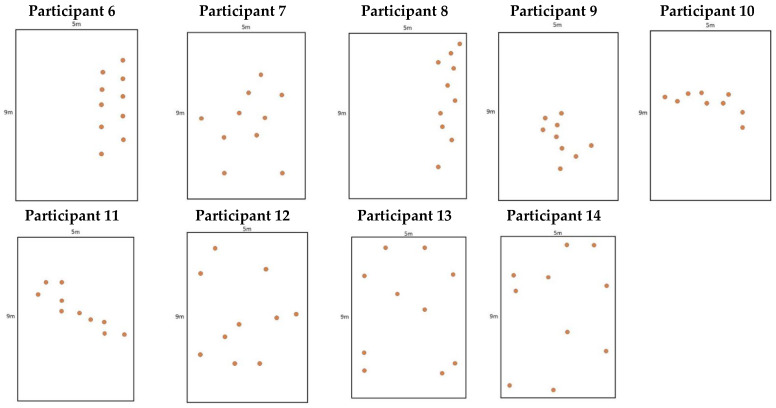
Individual dribbling patterns created by the participants.

The number of touches undertaken by children in the 1 min period was spread, ranging from 32 to 118. Pearson’s product moment correlations also revealed significant associations between the number of touches/min and average distance between cones (r = −0.671, *p* = 0.03), with a greater number of touches being associated with a smaller average distance between cones. There were also significant negative relationships between UGent dribbling test time and coach rating of dribbling ability (r = −0.67, *p* = 0.03) and total FMS (r = −0.746, *p* = 0.012), as well as between child perception of dribbling ability and total FMS score (r = 0.604, *p* = 0.049). All other relationships between the variables were non-significant (*p* > 0.05). Figure 1 presents the different patterns created by children as architects of the dribbling task.

**Figure 2:**
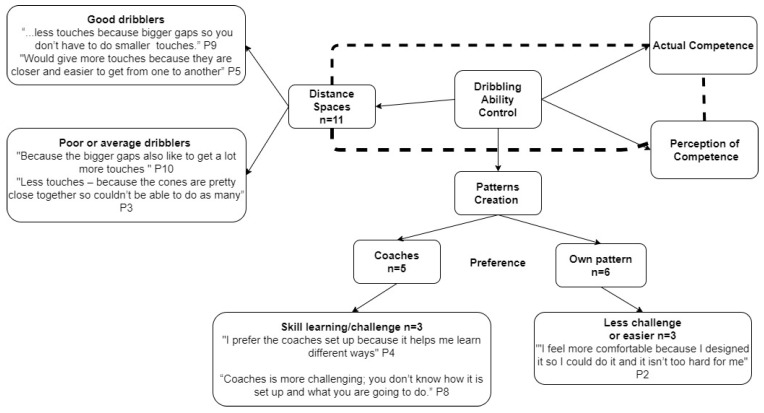
Pen profile diagram of children’s perceptions of the affordances for dribbling task.

With regard to the thematic analysis, three emergent but interconnected themes arose, focusing on: perception of ability and actual ability, and challenge and autonomy. Those children who perceived themselves as ‘good dribblers’ tended to create patterns where the cones were more closely spaced, while those who perceived themselves as poorer dribblers created patterns where the cones were more widely spaced (See Figure 2). The patterns of cones, which were more closely spaced, were created by the children to get more touches and make it more challenging as a task on the basis that this increased challenge resulted in better progress or development of the dribbling skill itself. Conversely, children who perceived themselves to be poor or average dribblers created patterns of cones with larger gaps to ensure they could ‘get a lot more touches’ while staying in control of the ball. When children were asked about their preference for developing dribbling skills using their own patterns or those provided by the coaches, there was a relatively even split between those who preferred the coach (*n* = 5) to create drills or those who would prefer autonomy to create the drills themselves (*n* = 6). Those whose preference was for self-created drills seemed to explicitly relate to challenge, for example: ‘because coaches have a lot of space between cones usually—these do not put the pressure on my dribbling and I would like to create challenge’ (P9) and: ‘*I feel more comfortable because I designed it so I could do it and it isn’t too hard for me’* (P2). Comments from the participants also related to having autonomy in practice, for example: ‘*If I need to work on something, I can do it as how I need to work on something’* (P10), and: ‘*Because it allows you to do what you would like to do with the ball, instead of [the coach], saying this is what you’re going to do with the ball’* (P11).

## 4. Discussion

The current study examined how children afford spaces for soccer dribbling using a constraints led approach. We uniquely examined children as architects of their soccer skill development in exploring how their perception of affordances for soccer dribbling influenced the number of touches on the ball in a soccer dribbling task. Understanding this process is important in developing practices to maximise soccer skill development for children and to inform coach practice related to player development. The results of the current study are congruent with the perspective that the environment consists of possibilities for action and is perceived as such, and in different ways by different children [2]. The present study suggests that those children who created patterns with less space between the cones accrued more touches of the football in their dribbling task. According to the affordances perspective, when asked to create a task, such as that used in the present study, a child would perceive the distance between two cones not in terms of a set distance but in terms of whether the gap between cones is sufficient for him/her to dribble through [2]. Thus, the current study suggests that affordances are not only primary in the children’s perception of the environment but also in the design of learning spaces and, if children can modify their environment, they do so in accordance with their perceived action capabilities for their physical ability and body [1].

The qualitative data suggest there is a feedback loop between a child’s perception of ability and their actual ability, which influences the types of dribbling patterns they create, which in turn relates to considerations by the children of challenge and autonomy. Those children who perceived themselves to be better dribblers created dribbling patterns that were more closely spaced, which, in turn, resulted in a greater number of touches on the ball. The creation of such patterns was anchored in the children wanting to challenge themselves in terms of their skill development and having the autonomy to create dribbling patterns to either challenge themselves, support themselves in gaining as many touches of the football as possible or to make the drill easier to ensure success. In this context, greater challenge via less space between cones ensured less time was afforded to move the ball between cones and thus required better performance of the motor skills to successfully do so.

Collectively, the results from this small-scale study suggest that engaging children as active participants in the creation of the dribbling drill appeared to reveal a discrepancy between the types of soccer dribbling typically employed in soccer coaching [18] and the patterns created by the children themselves. Such an observation is congruent with prior research investigating children’s active involvement in play space design [5] and is suggestive that historical design processes for movement, typically using standardisation of spaces [35], may be different when a child creates movement opportunities based on their (perceived) action capabilities [5]. Whether children engaging in soccer practice using practices they have wholly designed themselves results in greater skill development compared to practices prescribed by coaches would be an interesting next step arising from this exploratory study. Although we demonstrate that self-organisation of the cones by children increased the number of touches accrued in conditions of greater proximity, we did not examine whether this process might influence game performance. There is evidence, from a study of 15 male youth players, that team composition (e.g., fielding midfielders versus attackers) in small-sided games can influence the players’ capability of action that emerges during performance [36]. Whether self-organisation of practice in the way we operationalised in the present study translates into game performance is a natural progression from the findings of the present study. It is also important to note that different types of practice may be more or less important as a focus depending on the stage of development. While a blocked and constant approach to developing motor skill has been criticised [15], it may be useful in the first stages of fundamental development, while using a child-centred approach, such as that demonstrated in the current study, may be more beneficial once children have gone beyond the early development of soccer-specific motor skills. 

No study to date has examined how children’s perceptions of affordances affect the development of soccer skill, and, although exploratory, the current study’s approach, by using a mixed-methods design, represents an original contribution to the field. Using an experimental paradigm followed by a qualitative contextualisation of the affordance activity enables a richer understanding to be uncovered. The combination of quantitative and qualitative approaches in the present study is a novel contribution to the literature examining affordances for skill development in children’s soccer and in understanding how children structure space and work with constraints as architects of their own skill development.

We are cognisant that the results of the present study are based on a relatively small sample of grassroots footballers, all of whom were male. These should be considered limitations of the current study. Future work examining this process in girls and across different developmental stages of football skill is also needed to understand if involving children at earlier, or later, stages of skill development changes the way in which children afford dribbling in soccer. In addition, we did not assess biological maturation of the participants. It is possible that some of the sample participants were starting onset of growth spurt, given this is typically at around 12 years of age in boys [37], and our sample were aged 10–11 years. As biological maturation can change performance parameters, future research should consider the measurement of maturation when examining how children interact with the environment in sports-related circumstances. A key focus of the current study was in exploring whether the mixed-methods approach, employing an experimental design followed by qualitative exploration, adds value to scientific understanding of children’s soccer skill development. Additional research, using the approach employed in the current study, would therefore be useful in establishing the effectiveness of children as architects of their own skill development. The present study has some practical applications; notably, we demonstrate that coaches could empower children themselves to construct their own skill development practices for soccer, and, if they do, the children themselves are likely to scale their practices to their actual ability. This would suggest the co-creation of soccer practices between children and coaches could be an effective strategy for soccer skill development at grassroots level. Despite this, and to conclude, the current mixed-methods study demonstrates that children can create their own opportunities for skill development and that, when they do, these opportunities are related to their actual dribbling ability, reinforcing the affordances construct in practice. Dribbling performance is therefore scaled to the (perceived) action capabilities of the children, and children can act as architects in their own skill development.

## Figures and Tables

**Table 1:** Mean ± SD of touches/min, child perception of dribbling ability, time taken on the UGent dribbling test, total FMS score, coach rating of dribbling ability and the average distance between cones in each participant-created dribbling pattern.

Touches (No/Min)		Perceived Dribbling Ability (0–10)		UGent Dribbling Test (Secs)		Total FMS (0–54)		Coach Rating of Dribbling Ability (0–10)		Average Distance between Cones (cm)	
M	SD	M	SD	M	SD	M	SD	M	SD	M	SD
66.5	26.1	7.1	1.4	25.9	3.3	35.1	5.2	6.0	1.6	99.7	38.7

## Data Availability

Data supporting the results of the current study are available on request to the corresponding author.
